# Studying the Interplay Between Apolipoprotein E and Education on Cognitive Decline in Centenarians Using Bayesian Beta Regression

**DOI:** 10.3389/fgene.2020.606831

**Published:** 2021-01-08

**Authors:** Qingyan Xiang, Stacy Lynn Andersen, Thomas T. Perls, Paola Sebastiani

**Affiliations:** ^1^Department of Biostatistics, Boston University School of Public Health, Boston, MA, United States; ^2^Section of Geriatrics, Department of Medicine, Boston University School of Medicine, Boston, MA, United States; ^3^Institute for Clinical Research and Health Policy Studies, Tufts Medical Center, Boston, MA, United States

**Keywords:** apolipoprotein E, beta regression, blessed information-memory-concentration test, cognitive function, centenarians

## Abstract

Apolipoprotein E (*APOE*) is an important risk factor for cognitive decline and Alzheimer’s disease in aging individuals. Among the 3 known alleles of this gene: e2, e3, and e4, the e4 allele is associated with faster cognitive decline and increased risk for Alzheimer’s and dementia, while the e2 allele has a positive effect on longevity, and possibly on preservation of cognitive function. Education also has an important effect on cognition and longevity but the interplay between *APOE* and education is not well-characterized. Previous studies of the effect of *APOE* on cognitive decline often used linear regression with the normality assumption, which may not be appropriate for analyzing bounded and skewed neuropsychological test scores. In this paper, we applied Bayesian beta regression to assess the effect of *APOE* alleles on cognitive decline in a cohort of centenarians with longitudinal assessment of their cognitive function. The analysis confirmed the negative association between older age and cognition and the beneficial effect of education that persists even at the extreme of human lifespan in carriers of the e3 allele. In addition, the analysis showed an association between *APOE* and cognition that is modified by education. Surprisingly, an antagonistic interaction existed between higher education and *APOE* alleles, suggesting that education may reduce the positive effect of *APOE* e2 and increase the negative effect of *APOE* e4 at extreme old age.

## Introduction

Declines of certain cognitive abilities are common complications of aging and identifying risk factors for cognitive decline is essential to search for therapeutic interventions. Known risk factors for cognitive decline include older age, lower education, and genes such as apolipoprotein E (*APOE*) that plays an important role in the risk for Alzheimer’s disease (Fan et al., 2019). *APOE* is involved in the transport of cholesterol and other lipids between cellular structures (Mahley, 1988). The gene has three well-characterized alleles e2, e3, and e4 that are defined by the combinations of the genotypes of the single nucleotide polymorphisms rs7412 and rs429358. The three alleles differ in two amino acids and result in proteins produced by the e2 and e3 alleles that bind to high-density lipoprotein cholesterol (HDL) while e4 binds to very-low-density lipoprotein (VLDL) (Belloy et al., 2019). Studies have shown that e4-carriers have increased risk for Alzheimer’s disease and accelerated cognitive decline compared to non-carriers (Staehelin et al., 1999; Bondi et al., 2003; Wetter et al., 2005; Belloy et al., 2019), while e2-carriers appears to have a reduced risk for age-related neurodegenerative disease (Henderson et al., 1995; Raber et al., 2004; Kim et al., 2017). The review by O’donoghue et al. (2018) lists 40 studies of the association between *APOE* and cognition in longitudinal studies, but none of these studies examined this association among individuals at extreme ages (e.g., centenarians). Investigating the association of this gene with cognitive decline in centenarians is important for informing about the extent of the protective and deleterious effect of this gene on cognitive function at the extreme of human lifespan. In addition, examining the interaction between education and *APOE* alleles could help to better characterize the long term effect of education on cognition and the interplay between genetic and environmental risk factors. Therefore, in this work, we examined the association of e2 and e4 alleles of *APOE* with cognitive function in centenarians enrolled in the New England Centenarian Study (NECS) (Sebastiani and Perls, 2012), who are enriched for carriers of the e2 allele, have varying levels of education, and for whom we have longitudinally collected assessments of cognitive function.

Typically, longitudinal studies of the effect of *APOE* on cognitive decline use linear mixed models of data collected from a variety of neuropsychological tests (Blair et al., 2005; Caselli et al., 2009; Kim et al., 2017). These models rely on the assumption that errors follow a normal distribution, which is unbounded and symmetrically distributed. However, the outcome of any neuropsychological test can easily violate the normality assumption in two aspects: (1) the neuropsychological test scores are usually defined in a limited interval between 0 and a maximum test score, and (2) the distribution of neuropsychological test scores is often skewed. Violating the normality assumption when modeling cognitive test scores could lead to a poor estimation of the genetic effect on cognitive function, and predict scores that are either negative or exceed the maximum test value. To address this problem, we propose using a regression model based on the assumption that the response follows a beta distribution. Beta distribution is defined in a finite interval and can accommodate distribution with different shapes. Hence this distribution is well suited to model test scores and questionnaire outcomes that can take values in a finite range (Smithson and Verkuilen, 2006).

Beta regression was proposed by Ferrari and Cribari-Neto (2004) and was further developed by others (Smithson and Verkuilen, 2006; Simas et al., 2010; Figueroa-Zúniga et al., 2013). Studies in multiple fields have utilized beta regression to model different variables such as ischemic stroke lesion volume (Swearingen et al., 2011), genetic distance (Branscum et al., 2007), and understory vegetation communities (Eskelson et al., 2011). In this paper, we use a Bayesian hierarchical beta regression model to fit longitudinally collected neuropsychological test scores, and we compare the results of this analysis with the conventional linear mixed model. Using the Bayesian beta regression to analyze the association between *APOE* and cognitive decline among centenarians enrolled in the NECS is an important novelty of our analysis (Sebastiani and Perls, 2012).

## Materials and Methods

### Participants

The NECS is an ongoing study that began in 1994 as a population-based study of centenarians living within eight towns in the Boston area and expanded enrollment to include centenarians, their siblings and offspring as well as controls to North America in 2000. Enrolled participants provide socio-demographic data, medical history, and physical function ability (Barthel Index, Mahoney and Barthel, 1965; Sinoff and Ore, 1997). Participants are also administered the Blessed Information-Memory-Concentration (BIMC) test (Blessed et al., 1968; Kawas et al., 1995), which is a brief test of global cognition that can be administered over the phone to the participant or with the help of a proxy. The BIMC has a maximum total score of 37 points. Scores of 33 or greater represent no impairment, 27–32 indicate mild impairment, 21–26 signify moderate impairment, and less than 20 are associated with severe impairment (Kawas et al., 1995). NECS participants are followed annually to collect new medical events, changes in medication and physical function ability, and are administered the BIMC annually. In this analysis, we use the data collected through November 2019. The data include information about sex, education, race, ages, *APOE* genotypes, and BIMC scores of participants who agreed to complete the test. Participants with Alzheimer’s disease or dementia who could not complete the test were not included. Genotype data for *APOE* were inferred from the combinations of the SNPs s7412 and rs429358 as described in Sebastiani et al. (2019a).

### Statistical Analysis

#### Beta Regression Specification

A variable *y* defined in the interval (0, 1) follows the beta distribution if the density function is proportional to

$$f\left(y|\mu ,\phi \right)\propto y^{\mu \,\phi -1}{\left(1-y\right)}^{\left(1-\mu \right)\,\phi -1},$$

where μ represents the mean: 0 < *μ* < 1; the function *μ*(1−*μ*)/(1 + *ϕ*) is the variance, and *ϕ* > 0 is the precision parameter (Ferrari and Cribari-Neto, 2004). To parameterize the mean as a function of covariates, it is convenient to use the logit function

$$g\,\left(\mu \right)=l\,o\,g\,\left(\mu /\left(1-\mu \right)\right)=\,x_{\,i}^{\,T}\,\beta ,$$

where β is a vector of coefficient and $\,x_{\,i}^{\,T}$ is a vector of covariates (Smithson and Verkuilen, 2006; Zeileis et al., 2010). The beta distribution is defined in the open interval (0, 1). To fit this model to data defined in the range (*a*,*b*), we use the transformation *y*′ = (*y*−*a*)/(*b*−*a*). In our analysis, the BIMC scores range from 0 to 37. By adding/subtracting a small correct term from the minimum/maximum values of the BIMC scores, we rescaled the data to the interval [0.01, 0.99] to avoid zeros and ones.

#### Bayesian Beta Regression Modeling

We were interested in modeling the association of the following main covariates with BIMC scores: age, sex, education, and *APOE* alleles. We standardized the continuous variables *age* and *education* to generate parameters on the same scale and coded the dichotomous variable *sex* 0 for female and 1 for male. Since homozygote carriers of e2 or e4 are rare, we used the following allele groupings in the analyses:

1. “e2 group” comprising carriers of the *APOE* genotypes e2e2 or e2e3;
2. “e3 group” comprising carriers of the *APOE* genotype e3e3;
3. “e4 group” comprising carriers of the *APOE* genotypes e3e4 or e4e4.

We used dummy codings regarding these three groups, and we selected “e3 group” as the reference group in all of our analyses because it is the most frequent genotype in Whites. We performed two analyses to distinguish the beneficial effect of the e2 group allele relative to e3 group from lack of carrying the deleterious e4 allele. One analysis estimated the association between *APOE* and BIMC scores of the e2 group relative to the e3 group by including only the e2 groups and the e3 groups. The other analysis estimated the association between *APOE* and BIMC scores of the e4 group relative to the e3 group. For completeness, we also performed an additional analysis that included all three *APOE* groups.

We used backward selection for model fitting, and started with the hierarchical regression model:

$$y_{i\,j}∼\text{Beta}\,\left(\mu_{i\,j}\,\phi ,\phi \,\left(1-\mu_{i\,j}\right)\right)$$

$$\text{logit}\,\left(\mu_{i\,j}\right)=\mu_{b_{0}}\cdot I_{s\,i\,n\,g\,l\,e}+b_{i\,0}\cdot \left(1-I_{s\,i\,n\,g\,l\,e}\right)+\beta_{a\,g\,e}\cdot a\,g\,e_{i\,j}+$$

$$\beta_{a\,g\,e^{2}}\cdot a\,g\,e_{i\,j}^{2}+\beta_{s\,e\,x}\cdot s\,e\,x_{i}+\beta_{e\,d\,u}\cdot e\,d\,u_{i}+\beta_{A\,P\,O\,E}\cdot A\,P\,O\,E_{i}+$$

$$\beta_{a\,g\,e.e\,d\,u}\cdot a\,g\,e_{i}\cdot e\,d\,u_{i}+\beta_{a\,g\,e.A\,P\,O\,E}\cdot a\,g\,e_{i}\cdot A\,P\,O\,E_{i}+$$

$$\beta_{e\,d\,u.A\,P\,O\,E}\cdot e\,d\,u_{i}\cdot A\,P\,O\,E_{i},$$

where *y*_*ij*_ denotes the *j*^*th*^ cognitive test score of the *i*^*th*^ participant, the β coefficients are fixed effects, and *b*_*i0*_*b*_*i0*_ is the random intercept that we used to account for within participant correlation of the repeated measurements. Besides the covariates of the main effects of age, sex, education, and *APOE*, we also included a squared term of age and two-way interactions of these main effects in our model. We used a piecewise random intercept *μ*_*b*__0_⋅*I*_*single*_ + *b*_*i*0_⋅(1−*I*_*single*_) to accommodate participants with different number of test administration, where the indicator variable *I*_*single*_ is 0 for participants with only one cognitive test score and *I*_*single*_ = 1 otherwise. The random intercepts *b*_*i0*_ were assumed to be independent and normally distributed, i.e., *b*_*i0*_∼*N*(*μ*_*b*_,σ^2^_*b*_). We specified a normal prior for the mean parameter *μ*_*b*_ of the random intercept that *μ*_*b*_∼*N*(0, 1000), and a gamma distribution with the shape and scale parameters both equal to 1 for the precision parameter ϕ and the precision of random intercept 1/σ^2^_*b*_. With this parameterization, the participants with only one test score would be assigned the fixed intercept *μ*_*b*_ in the regression, while participants with more than one test score have their own random intercept *b*_*i*0_. We used normal priors for all the fixed effect parameters.

We implemented the backward model selection algorithm using the deviance information criterion (DIC) (Spiegelhalter et al., 2002, 2014), which is particularly useful for selection of hierarchical models. Since DIC has a tendency to overfit (Clarke and Clarke, 2018), we then refined the model selected by this search by retaining only interactions and main effects with a posterior credible interval (CI) that did not include 0. Once we selected the final model, we also conducted a sensitivity analysis with respect to the prior distributions. For regression coefficients and the mean parameter of the random intercept that use the normal priors, we reduced the variance from 1,000 to 10 and 100. For precision parameters of the beta distribution and the random intercept that use the gamma priors, we modified the variance of gamma priors from 1 to 100. We ran each case to assess if the parameter estimates altered after we changed the scale of the prior parameters. We estimated the explained variance of the model using the regression sum of squares divided by the total sum of squares. All analyses were conducted in R3.6 and all Bayesian models were analyzed using Markov Chain Monte Carlo (MCMC) implemented in the “rjags” package (Plummer, 2016). The posterior estimates of the parameters are derived from at least 8,000 burn-in adaptions and 4,000 iterations.

#### Interpretation of the Results

A limitation of beta regression is that the magnitude of the regression coefficients is not directly interpretable in terms of changes of the outcome. To better understand the association of *APOE* with BIMC scores and the interplay with education, we estimated the fitted means (marginal effect) using inverse transformation of the logit function, and then we rescaled the fitted means to the original scale of the BIMC scores (0, 37). We calculated fitted means for the e2, e3, and e4 groups for three different education levels: low education (25% quantile of study population’s education; 8 years), median education (median of study population’s education; 12 years), and high education (75% quantile of study population’s education; 15 years). For each allele in each education level, we also calculated the corresponding age of onset of moderate cognitive impairment (BIMC score = 26) using the fitted trajectories of the cognitive test scores. These ages provide a quantitatively more interpretable metric of the genetic effects.

#### Comparison With the Conventional Method

For comparison with the conventional method, we performed two analyses that focus on the effect of e2 or e4 separately using linear mixed models. For a fair comparison, we used the same variables as selected by Bayesian beta regression, and we kept the random intercept term to adjust for the subject effect. The linear mixed model was conducted in R3.6 using the package “lme4.” We visualized the results of these two methods by plotting the fitted BIMC scores (marginal effect) with their credible/confidence intervals against age. In the Bayesian beta regression, the credible interval of the fitted value was computed using the 2.5th and 97.5 percentiles from the MCMC samples. In the linear mixed model, the confidence interval of the fitted values was derived using bootstrap. We also computed a residual sum of squares (RSS) for each method.

## Results

### Applying Bayesian Beta Regression on the NECS

Out of 768 total participants in the NECS dataset with *APOE* genotypes, we excluded 167 participants with missing test scores, 111 participants with missing education information, and 4 participants with *APOE* genotype e2e4. Table 1 summarizes the demographic characteristics and test scores at baseline of the 486 participants used in our analysis. The age range of the data used in our analysis was 91–113 years. The *APOE* e3 group was the most prevalent and used as the reference group in all subsequent analyses. Approximately 24% of centenarians in the study carried at least one e2 allele, while only 8% were carriers of an e4 allele. We did not find any e4 homozygous centenarians.

**TABLE 1:** Demographic characteristics and the BIMC scores at baseline of the participants in the New England Centenarian Study.

	***APOE* e2 (e2e2, e2e3)**	***APOE* e3 (e3e3)**	***APOE* e4 (e3e4)**
N at enrollment	117	331	38
N with at least 2 follow-ups	60	170	20
Sex, male (%)	22 (18.8%)	85 (25.68%)	9 (23.68%)
**Age at enrollment**			
91–95 (%)	4 (3.42%)	10 (3.02%)	2 (5.26%)
96–100 (%)	22 (18.80%)	55 (16.61%)	9 (23.68%)
101–105 (%)	30 (25.64%)	94 (28.40%)	10 (26.32%)
106–110 (%)	50 (42.74%)	152 (45.92%)	16 (42.10%)
111-113 (%)	11 (9.40%)	20 (6.04%)	1 (2.63%)
Mean (SD)	103.54 ± 4.59	103.32 ± 4.43	102.42 ± 4.97
Years of Education, mean (SD)	11.63 ± 3.51	11.82 ± 3.85	12.29 ± 4.05
**BIMC scores at baseline**			
33–37 (%)	29 (24.79%)	98 (29.61%)	12 (31.58%)
27–32 (%)	33 (28.21%)	66 (19.94%)	7 (18.42%)
21–26 (%)	30 (25.64%)	91 (27.49%)	8 (21.05%)
0–20 (%)	25 (21.37%)	76 (22.96%)	11 (28.95%)
Mean (SD)	25.11 ± 8.23	25.00 ± 8.63	24.38 ± 10.20

*The percentiles are with respected to each column stratum. The groups of the BIMC scores are used to classify the degree of cognitive impairment: normal, 33–77; mild, 27–32; moderate, 21–26; severe, 0–21.*

**FIGURE 1:**
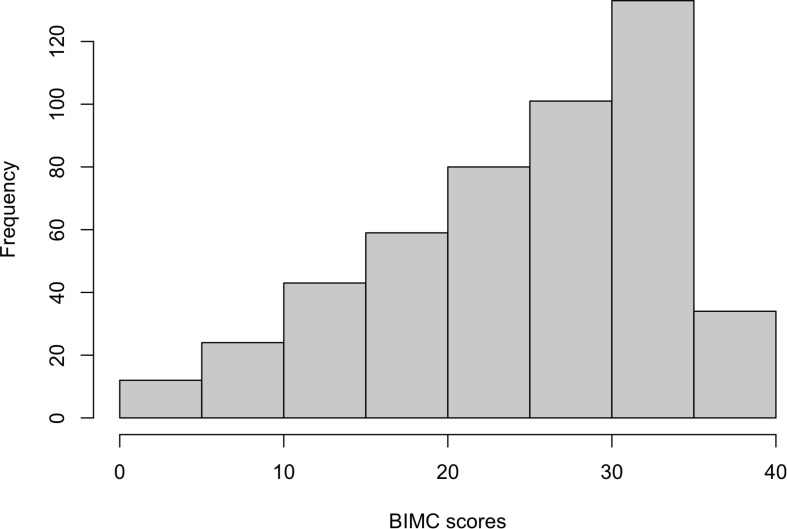
Histogram of baseline Blessed Information-Memory-Concentration scores from the New England Centenarian Study.

The histogram in Figure 1 shows that the distribution of the baseline BIMC scores is bounded and highly skewed. Hence we analyzed the rescaled test scores using beta regression. The combination of backward selection using the DIC criterion and refinement of the model using the posterior credible intervals produced the models that are summarized in Tables 2, 3. The diagnostic plots (trace plots, autocorrelation plots, and the Gelman plots of all MCMC chains) showed no indications of lack of convergence. The sensitivity analyses also showed that changing the prior distributions did not appreciably alter the parameter estimates. The diagnostic plots and sensitivity analyses are all included in Supplementary Material.

**TABLE 2:** Parameter estimates and 95% credible intervals from the analysis of the BIMC scores in carriers of *APOE* e2 and e3 alleles using Bayesian beta regression.

	**Estimates**	**2.5% CI**	**97.5% CI**	**SD**
Intercept (μ_*b*_)	**0.592**	**0.489**	**0.695**	**0.053**
Age	**−0.110**	**−0.138**	**−0.099**	**0.009**
Sex, male	**0.262**	**0.076**	**0.447**	**0.096**
Education	**0.062**	**0.039**	**0.085**	**0.012**
*APOE*2	0.037	**−**0.162	0.186	0.088
*APOE*2*edu	**−0.063**	**−0.109**	**−0.019**	**0.023**

*All covariates were standardized and the score was rescaled to the interval (0, 1) to fit the beta regression, so all parameters should be interpreted on the logit scale and the effects are relative to the rescaled scores. Bold fonts highlight significant parameters.*

**TABLE 3:** Parameter estimates and 95% credible intervals from the analysis of the BIMC scores in carriers of *APOE* e4 and e3 alleles using Bayesian beta regression.

	**Estimates**	**2.5% CI**	**97.5% CI**	**SD**
Intercept (μ_*b*_)	**0.647**	**0.541**	**0.685**	**0.053**
Age	**−0.119**	**−0.141**	**−0.098**	**0.011**
Sex, male	**0.215**	**0.019**	**0.413**	**0.101**
Education	**0.065**	**0.041**	**0.089**	**0.012**
*APOE*4	**−0.382**	**−0.664**	**−0.103**	**0.139**
*APOE*4*edu	**−0.082**	**−0.159**	**−0.008**	**0.034**

*All covariates were standardized and the score was rescaled to the interval (0, 1) to fit the Bayesian beta regression, so all parameters should be interpreted on the logit scale and the effects are relative to the rescaled scores. Bold fonts highlight significant parameters.*

Table 2 summarizes the results of the analysis comparing *APOE* e2 with *APOE* e3, and Table 3 summarizes the results of the analysis comparing *APOE* e4 with *APOE* e3. The significant coefficients are marked with bold fonts. In both analyses, age, sex, and education were all significantly associated with BIMC scores. Older age was associated with significantly worse performance on the test. More years of education and male sex were associated with significantly higher scores.

**FIGURE 2:**
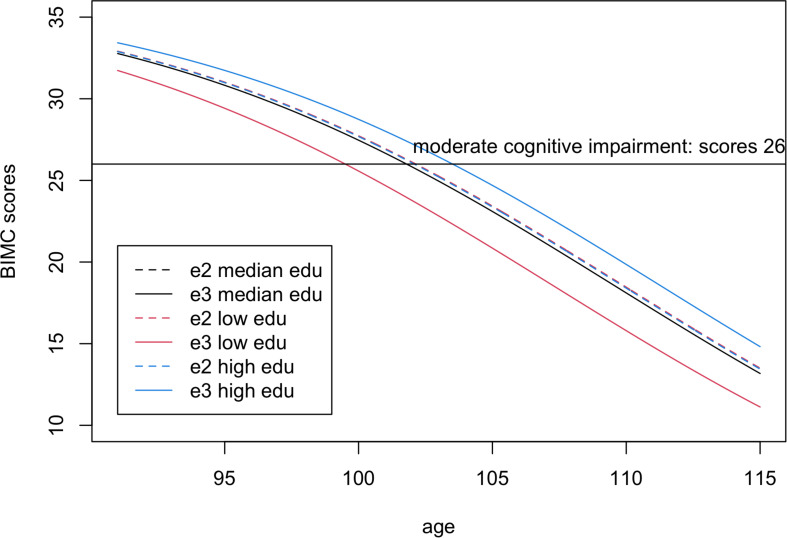
Fitted Blessed Information-Memory-Concentration scores in carriers of one or more e2 alleles (dashed lines) compared to homozygotes e3e3 (continuous lines) for low education level (25% quantile, 8 years, red), median education level (12 years, black), and high education level (75% quantile, 15 years, blue). The scores were fitted using the estimates from Bayesian beta regression, assuming sex = female. Note visually there is only one dashed line, because the fitted lines of e2 carriers overlap in all three education groups.

In the analysis comparing e2 with e3, the interaction between education and e2 was significant, thus suggesting an effect modification of education on the association of e2 with BIMC scores. It is noteworthy that the estimates of the main effect of education (0.063) and the interaction term (−0.062) were opposite, so that the negative interaction between e2 and education numerically canceled out the positive effect of education on BIMC scores in carriers of the e2 allele. This is shown in Figure 2, in which the fitted BIMC scores in e2 carriers remain nearly the same in different education levels. Figure 2 also shows the positive effect of education on BIMC scores in the e3 group. Interestingly, only participants of the e3 group with low education had a worse BIMC score compared to carriers of one or more e2 alleles. The difference between the e2 and e3 groups became negligible in participants with median education level, and carriers of the e3e3 genotypes with a high level of education obtained a better score than carriers of the e2 alleles.

To better summarize the clinical implication of these results, we calculated the ages of onset of cognitive impairment predicted by the fitted model. From the fitted lines, we estimated that the ages of onset of moderate cognitive impairment were 99.5, 101.8, and 103.5 years in for e3 carriers with low, median, and high education groups, respectively. The age of onset of moderate cognitive impairment (BIMC score = 26) was 102.1 years in e2 carriers, independent of education. Therefore, e2 carriers were estimated to delay the onset of moderate cognitive impairment by approximately 2 years compared to e3 carriers with low education, but this advantage essentially disappeared with higher education.

**FIGURE 3:**
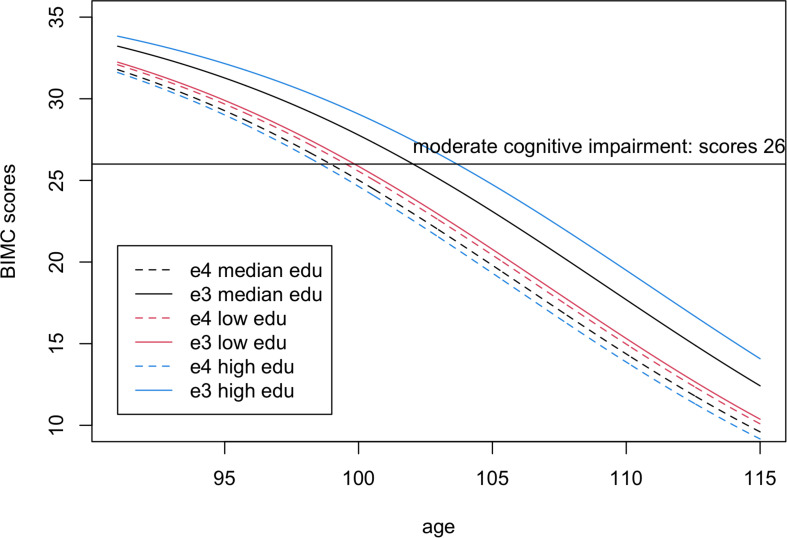
Fitted Blessed Information-Memory-Concentration test scores in carriers of one or more e4 alleles (dashed lines) compared to homozygotes e3e3 (continuous lines) for low education level (25% quantile, 8 years, red), median education level (12 years, black), and high education level (75% quantile, 15 years, blue). The scores were fitted using the estimates from Bayesian beta regression, assuming sex = female.

In the analysis comparing e4 with e3, both the main effect term of e4 and the interaction with education were significantly negative, thus suggesting that higher education was not sufficient to remove the negative association of the e4 allele with BIMC scores. This is illustrated in Figure 3 that shows the fitted BIMC scores in the e3 and e4 groups stratified by education. Among individuals with the e3e3 genotype, more years of education was associated with higher BIMC scores, but the positive effect modification of higher education was reduced in carriers of e4 compared to e3.

From the fitted lines, the ages of onset of moderate cognitive impairment in e4 carriers were 99.5, 98.9, and 98.57 years in participants with low, median, and high education, respectively. Hence, in centenarians carrying the e4 allele, more years of education was not associated with a delay of cognitive decline.

The results of the combined analysis including all three groups of *APOE* alleles are summarized in Supplementary Table 1. After using the same model selection strategy as the previous separated analyses, the selected model had exactly the same variables of the two separated analyses. The direction of the effects of *APOE* and the significance of the variables were consistent with the previous analyses. The major difference was that the magnitude of the effects of *APOE* decreased in the combined analysis. For example, in the separated analysis, the beta coefficients of e2 and e4 were 0.032 and −0.382, respectively, while in the combined analysis the beta coefficients of e2 and e4 were 0.026 and −0.326. Hence, the analysis that combined all *APOE* alleles together suggested slightly smaller but still significant effects of *APOE.*

### Comparison With Conventional Linear Mixed Models

The results of the analyses using linear mixed models are summarized in Tables 4, 5. In both analyses, only the effects of age and education reached statistical significance. The direction of the main effects of e2 and e4 were consistent with those estimated using the Bayesian beta regression. However, the effects did not reach statistical significance.

**TABLE 4:** Parameter estimates from the analysis of the BIMC scores in carriers of *APOE* e2 and e3 alleles using the linear mixed model.

	**Estimates**	**SD**	***T*-value**	***P*-value**
Intercept	**23.935**	**0.474**	**50.544**	**< 0.001**
Age	**−1.074**	**0.064**	**−16.844**	**< 0.001**
Sex, male	1.251	0.859	1.457	0.146
Education	**0.438**	**0.109**	**4.032**	**< 0.001**
*APOE2*	0.075	0.82 0	0.092	0.927
APOE2*edu	−0.430	0.229	−1.876	0.061

*Bold fonts highlight significant parameters.*

**TABLE 5:** Parameter estimates from the analysis of the BIMC scores in carriers of *APOE* e4 and e3 alleles using the linear mixed model.

	**Estimates**	**SD**	***T*-value**	***P*-value**
Intercept	**24.386**	**0.495**	**49.233**	**< 0.001**
Age	**−1.082**	**0.071**	**−15.174**	**< 0.001**
Sex, male	1.073	0.940	1.142	0.254
Education	**0.437**	**0.111**	**3.934**	**< 0.001**
*APOE4*	−2.286	1.330	−1.719	0.087
APOE4*edu	−0.485	0.333	−1.458	0.146

*Bold fonts highlight significant parameters.*

**FIGURE 4:**
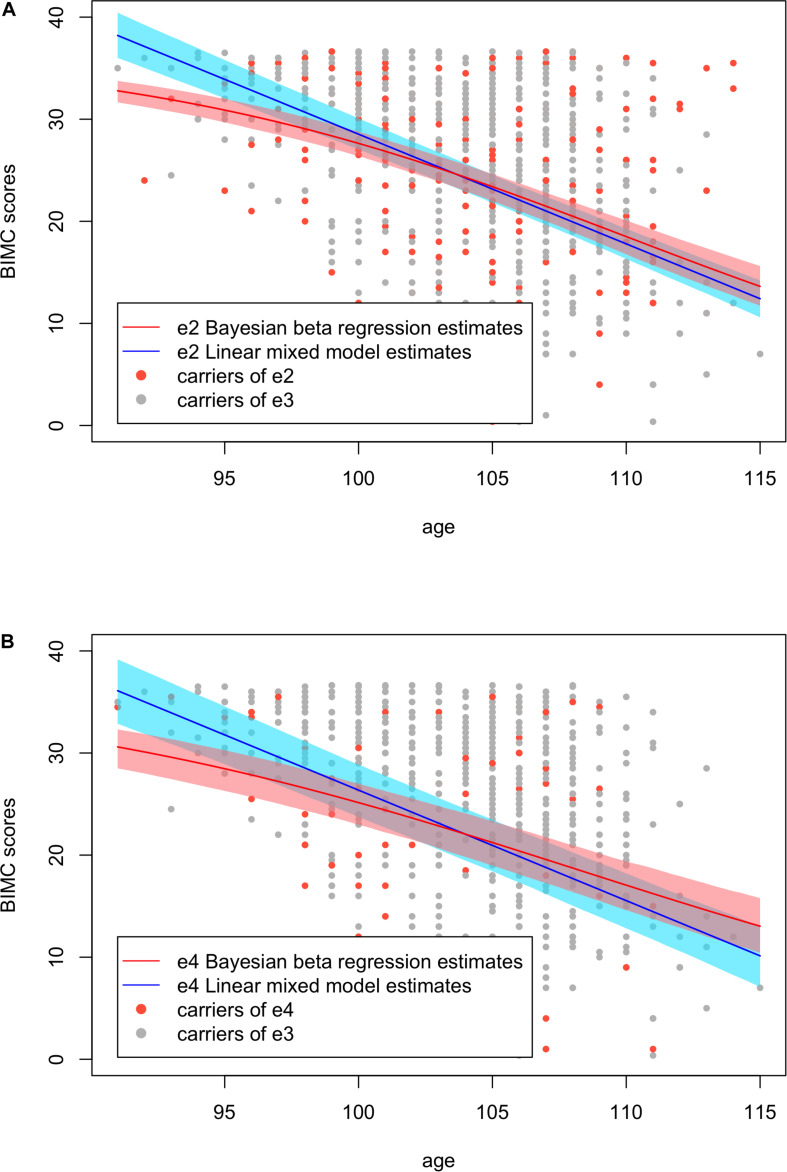
Fitted Blessed Information-Memory-Concentration test scores using Bayesian beta regression and the linear mixed models with their credible/confidence bands. **(A)** Analysis in carriers of *APOE* e2 and e3. **(B)** Analysis in carriers of *APOE* e4 and e3.

To visualize the differences of the results from Bayesian beta regression and the linear mixed model, we plotted the fitted BIMC scores with their 95% credible/confidence intervals against age in Figure 4. The plot shows that the credible intervals of the Bayesian beta regression were slightly narrower compared with the confidence intervals of the linear mixed model. The linear mixed model did not fit the data of the youngest age group well, and the fitted values as well as their confidence interval were even greater than the maximum BIMC score (max score = 37). This result is consistent with our hypothesis that using a linear mixed model with normality assumptions is not suitable when the outcomes are bounded neuropsychological scores. In addition, the Bayesian beta regression appeared to better capture the gradually increasing rate of decline, but the linear mixed model did not capture this feature. The RSS of the Bayesian beta regression was 51032.5 for the e2 analysis and 43151.54 for the e4 analysis, while the RSS of the linear mixed models was slightly larger (52443.72 for the e2 analysis and 43652.30 for the e4 analysis), thus confirming that the Bayesian beta regression fit the data better.

## Discussion

In this paper, we investigated the relationship between *APOE* alleles and change of cognitive function in a large cohort of centenarians enriched for carriers of the e2 allele. Instead of using the conventional linear mixed model, we used a Bayesian hierarchical beta regression model to characterize the effect of *APOE* on cognitive function assessed through the Blessed Information-Memory-Concentration test. Our analyses confirmed the decline of cognitive function and the positive effect of education on preservation of cognitive function at extreme old age. The analysis also showed that the *APOE* e4 allele has a negative association with cognitive function even at extreme old age, and the *APOE* e2 allele appears to be protective only in centenarians with low education.

Our findings are consistent with previous studies that showed a decline of cognitive function with older age in multiple domains (Harada et al., 2013), and the negative effect of *APOE* e4 on cognitive decline in centenarians (Arosio et al., 2017; Du et al., 2020). There is some evidence that, among centenarians, the e2 allele confers protection from cognitive decline (Kim et al., 2017; Sebastiani et al., 2020), in addition to increasing the chance for longevity (Sebastiani et al., 2019a) and conferring protection from aging-related diseases (Wolters et al., 2019; Kuo et al., 2020). Our studies also found that carriers of e2 with low education can delay their onset of moderate cognitive impairment. These results suggest that targeting e2 gene products could lead to important therapeutics for the preservation of cognitive function (Sebastiani et al., 2019b). Interestingly, the persistence of the e4 allele in the population has been linked to a survival advantage at a young age and treatments that target the effect of *APOE* alleles at an older age will need to consider the pleiotropic effect of this gene (Belloy et al., 2019).

Studies have shown the importance of early education on better cognitive function at an older age (Alley et al., 2007; Wilson et al., 2009). However, our study showed an antagonistic interaction between higher education and *APOE* alleles that suggests higher education may reduce the positive effect of e2 and increase the negative effect of e4 at extreme old age. A study of Japanese centenarians detected an education by *APOE* e4 interaction on cognition that differed by sex (Ishioka et al., 2016). The study showed that highly educated centenarians who carried at least one e4 allele had worse performance in the Mini-Mental State Examination, compared to poorly educated centenarians. Our findings are consistent with this counter-intuitive result but go one step further and disentangle the negative association of the e4 allele from the positive association of the e2 allele.

Our study focused on the association between *APOE* alleles and cognitive decline in extreme old individuals. Our analysis included well-known risk factors of cognitive decline such as older age and education. However, many more factors may affect the onset and rate of cognitive decline together or independently of *APOE*. Nutrition and dietary habits, for example, may be important risk factors to be considered in future analyses, given the role of *APOE* in lipid metabolism (Belloy et al., 2019). Studies showed that place of living changes the association between *APOE* alleles and extreme human longevity, after controlling for the overall genetic background (Gurinovich et al., 2019), suggesting that lifestyle may modify the genetic effect of *APOE*. However, we do not have data about dietary patterns in NECS centenarians to perform these analyses. The explained variance of our model was 0.29 for the e2 analysis and 0.30 for the e4 analysis. These results suggest that age, sex, *APOE*, and education can only explain less than 1/3 of the variability in BIMC scores and there are many factors, yet to be discovered, that should account for the unexplained variance. In future work, it will be interesting to collect data about additional physiological, medical, nutritional, or social factors that may contribute to maintain good cognitive function as people age and investigate their interplay with *APOE* alleles.

The Bayesian beta regression we used in this analysis has several strengths. First, this method allowed us to fit the test scores in the range of admissible values, and to model non-linear relations of the score with age and education. We illustrated the advantages of Bayesian beta regression by comparing it with the conventional linear mixed model using the NECS data. Second, to increase statistical power, we included all participants with at least one cognitive function assessment in our model. A piecewise random intercept was used to include subjects with either repeated measurements or just one measurement. Finally, we carried out a model selection using the DIC criterion and then refined our model by credible intervals. From the diagnostic plots of the MCMC, the final models converged well. In addition, in previous studies (Caselli et al., 2009; Kulminski et al., 2015; Barral et al., 2017) *APOE* genotype was characterized as carriers of e4 (i.e., e2e4, e3e4, and e4e4), and non-carriers of e4 (i.e., e2e2, e2e3, and e3e3). This comparison could yield biased results since the non-carriers of the e4 group might not represent the most prevalent population. There are also studies (Kim et al., 2017) included e2 (e2e2 and e2e3), e3 (e3e3), and e4 (e3e4 and e4e4) in a combined analysis with dummy codings. In fact, we performed a combined analysis on the NECS data and found that it produced smaller effects of e2 and e4. To eliminate these potential bias, we divided the participants into subgroups, where we only compared e2 carriers to e3 carriers and e4 carriers to e3 carriers separately.

Notable limitations of this study include loss to follow-up and mortality. Due to the extreme age of the study population, the mortality rate is considerably high: 49% of participants included in our analysis only completed the baseline evaluation. The absence of longitudinal data may induce bias, and thus we used a piecewise random intercept to compensate for those only with one record.

In conclusion, we examined the association between *APOE* alleles and cognitive decline in a cohort of centenarians. We confirmed the negative correlation between the e4 allele and cognition even at the extreme of human lifespan, and newly found the carrying the e2 alleles appears to be beneficial only in centenarians with poor education. The interaction between *APOE* e4 and education produced a counter-intuitive result that is, however, consistent with other results in centenarians. The antagonistic relation between higher education and carrying the e4 allele may be confounded by other risk factors and warrants more in-depth studies.

## Data Availability Statement

The data analyzed in this study is subject to the following licenses/restrictions: The use of the data is restricted and needs approval. Requests to access these datasets should be directed to PS, psebastiani@tuftsmedicalcenter.org.

## Ethics Statement

The studies involving human participants were reviewed and approved by the Boston University IRB. The patients/participants provided their written informed consent to participate in this study.

## Author Contributions

QX proposed the method used in the manuscript, conducted the statistical analysis, and drafted the manuscript. SA and TP designed the study, collected the data from the study cohort, and helped editing the manuscript. PS designed the analytic strategy, supervised the work, and critically revised the manuscript. All authors contributed to the article and approved the submitted version.

## Conflict of Interest

The authors declare that the research was conducted in the absence of any commercial or financial relationships that could be construed as a potential conflict of interest.
