# Supplementary material for: Studying the Interplay Between Apolipoprotein E and Education on Cognitive Decline in Centenarians Using Bayesian Beta Regression
Source: Front Genet. 2021 Jan 8;11:606831. doi: 10.3389/fgene.2020.606831 (PMC7820893; doi:10.3389/fgene.2020.606831)
Supplement: Supplementary file 1 [file Data_Sheet_1.docx]

Supplementary Material

# Supplementary analysis

The results of combined analysis including all three groups of *APOE* alleles are summarized in supplementary table 1. The significant variables are marked in bold. After using the same model selection strategy as the separated analyses, the selected model had exactly the same variables of two separated analyses: the main effect of age, sex, education, and *APOE*, and the interaction effect between *APOE* and education. The direction of the effects of *APOE* and the significance of the variables were consistent with the separated analyses described in the article.

The major difference in the results was that the absolute values of the coefficients of e2 and e3 in the combined analysis were smaller than the absolute values of coefficients in each separated analysis. For example, in the separated analysis, the beta coefficients of e2 and e4 were 0.037 and -0.382 respectively, while in the combined analysis the beta coefficients of e2 and e4 were 0.026 and -0.326. The magnitude of the effects of *APOE* decreased in the combined analysis. Hence, for the e2 and e4 that have an opposite effect on cognitive decline, the analysis combining all *APOE* alleles together suggested slightly smaller although still significant effects of *APOE*.

**Supplementary Table 1.** Parameter estimates and 95% credible intervals from the analysis of the BIMC scores in carriers of all three *APOE* alleles

|  | Estimates | 2.5%CI | 97.5%CI | SD |
| --- | --- | --- | --- | --- |
| Intercept ($\mu_{b})$ | **0.597** | **0.500** | **0.697** | **0.050** |
| Age | **-0.106** | **-0.124** | **-0.088** | **0.009** |
| Sex, male | **0.326** | **0.142** | **0.490** | **0.090** |
| Education | **0.063** | **0.040** | **0.083** | **0.011** |
| *APOE*4 | **-0.326** | **-0.607** | **-0.065** | **0.138** |
| *APOE*4*edu | **-0.077** | **-0.139** | **-0.005** | **0.034** |
| *APOE2* | 0.026 | -0.133 | 0.202 | 0.087 |
| *APOE2**edu | **-0.064** | **-0.110** | **-0.016** | **0.024** |

All covariates were standardized and the score was rescaled to the interval (0, 1) to fit the beta regression, so all parameters should be interpreted on the logit scale and the effects are relative to the rescaled score.

# Supplementary Figures and Tables

## Supplementary Figures


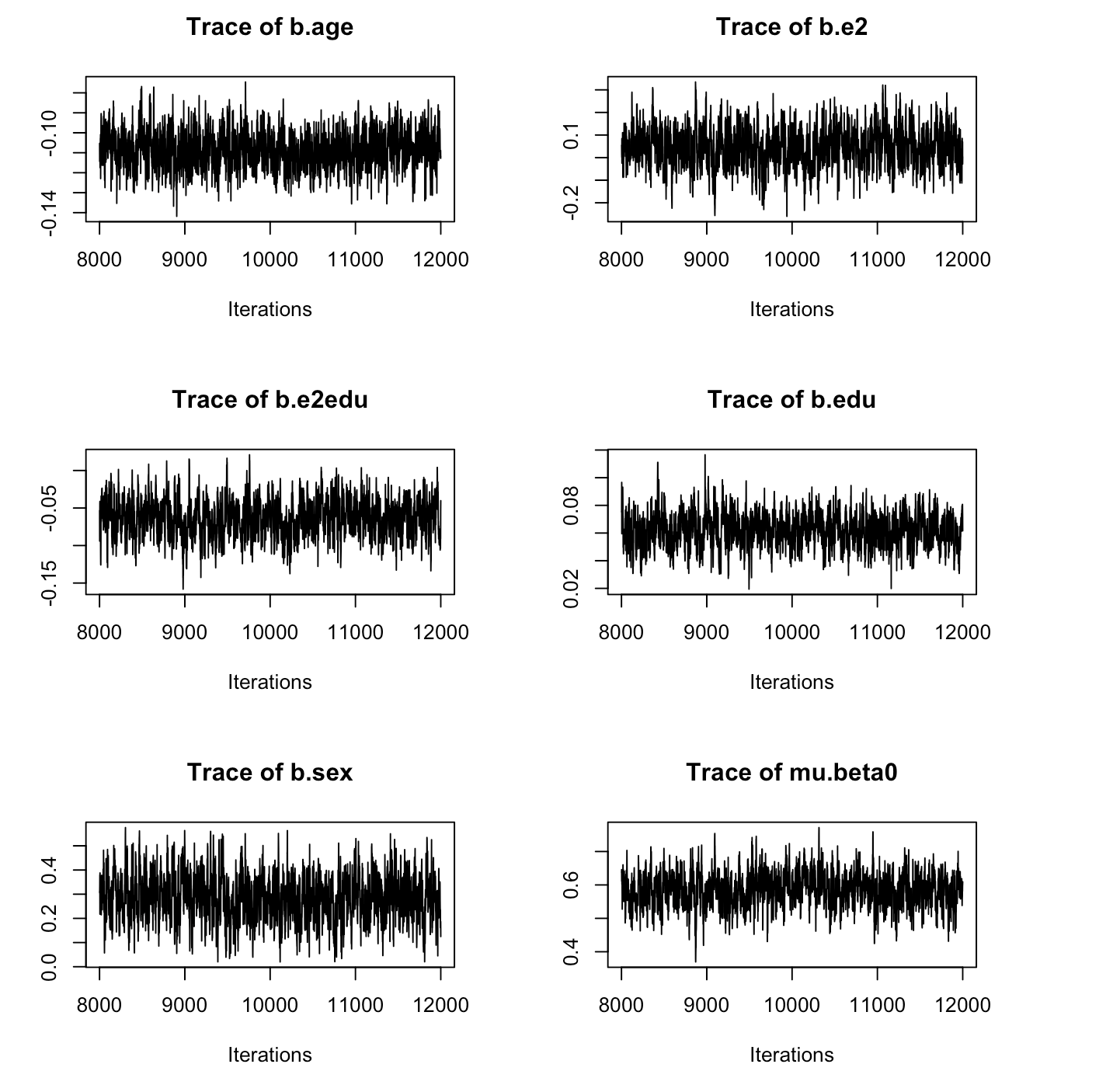


**Supplementary Figure 1**. Trace plot from the analysis of BIMC scores in carriers of *APOE* e2 and e3 alleles


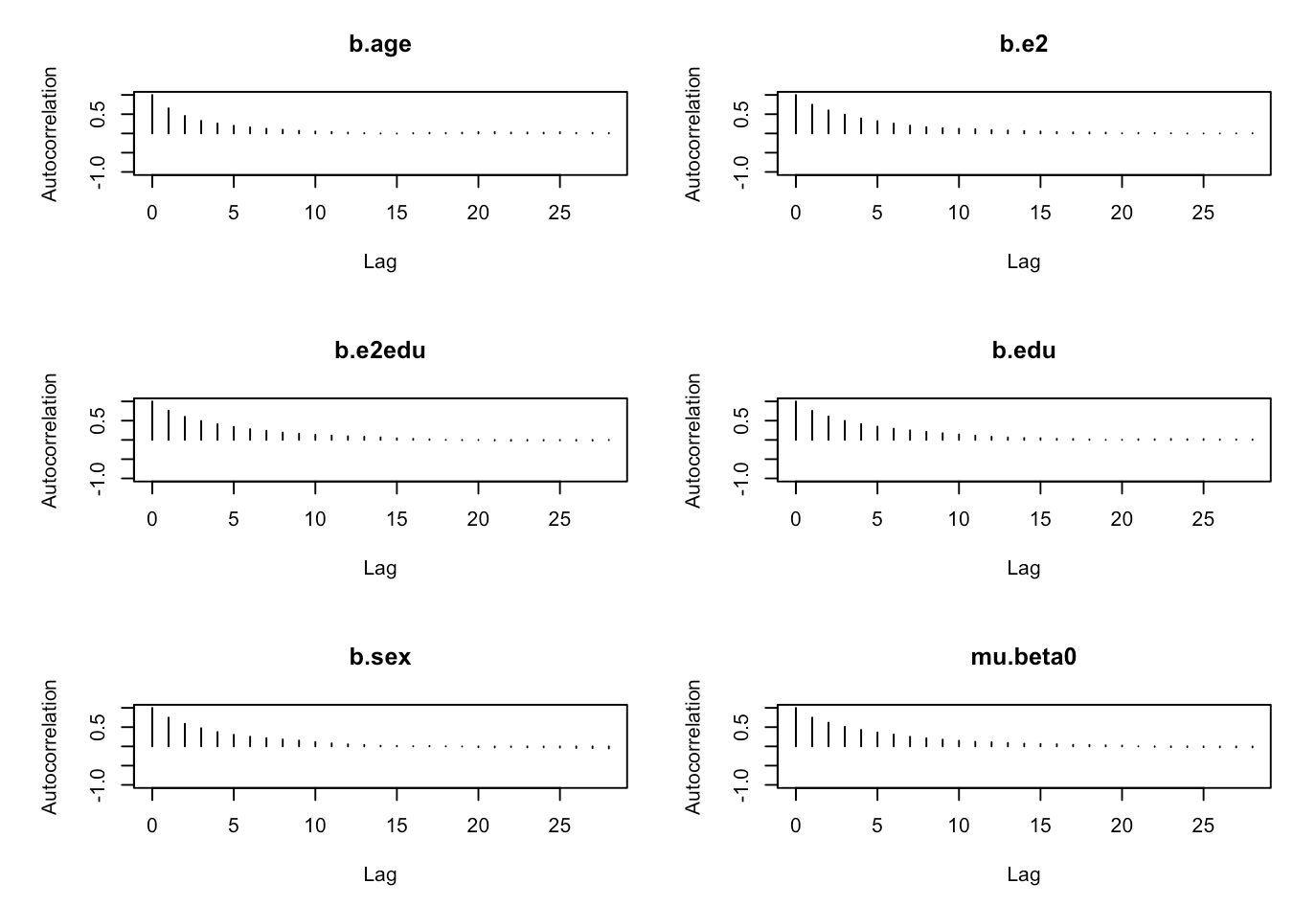


**Supplementary Figure 2**. Autocorrelation plot from the analysis of BIMC scores in carriers of *APOE* e2 and e3 alleles.


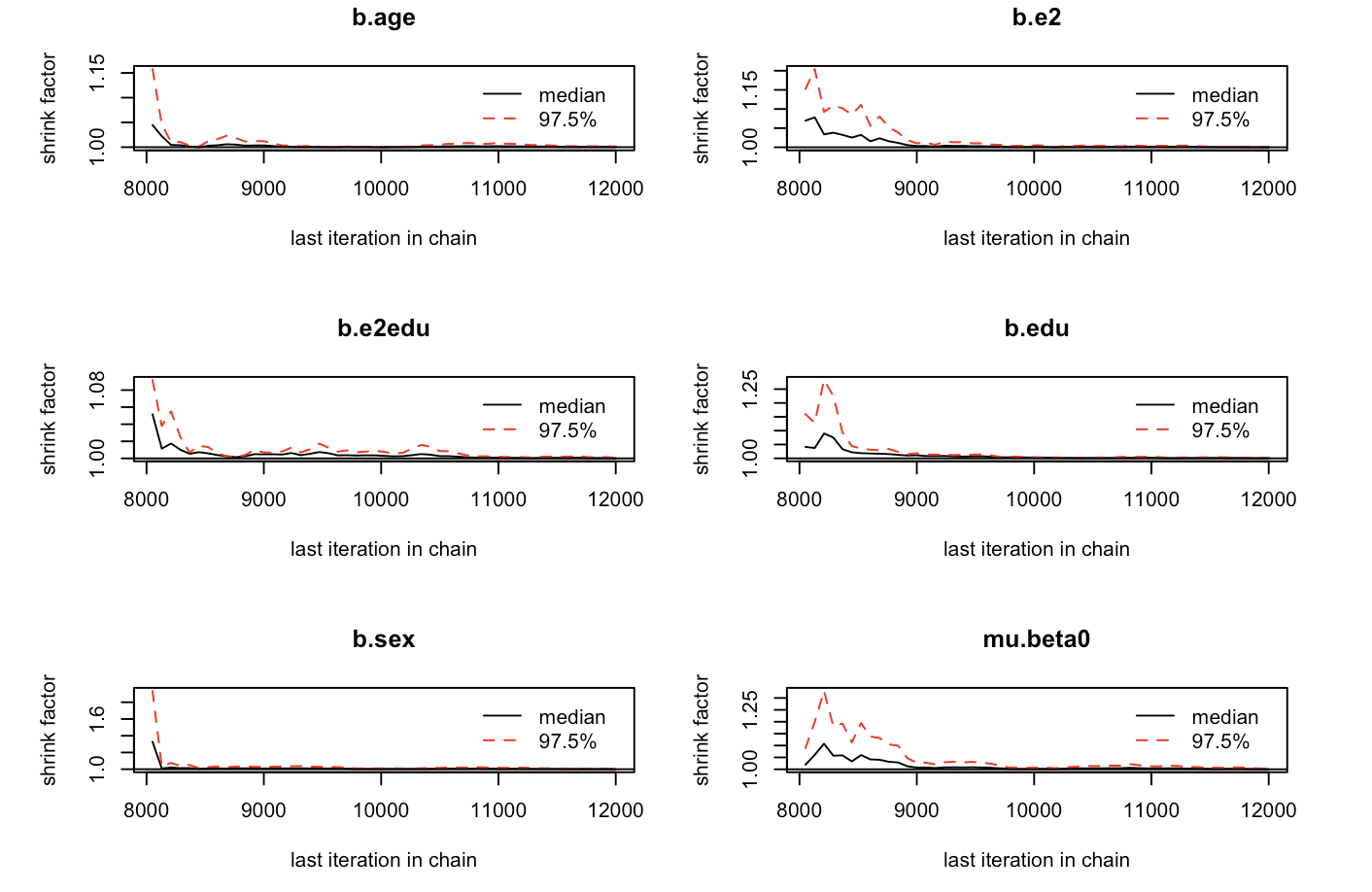


**Supplementary Figure 3**. Gelman plot from the analysis of BIMC scores in carriers of *APOE* e2 and e3 alleles.


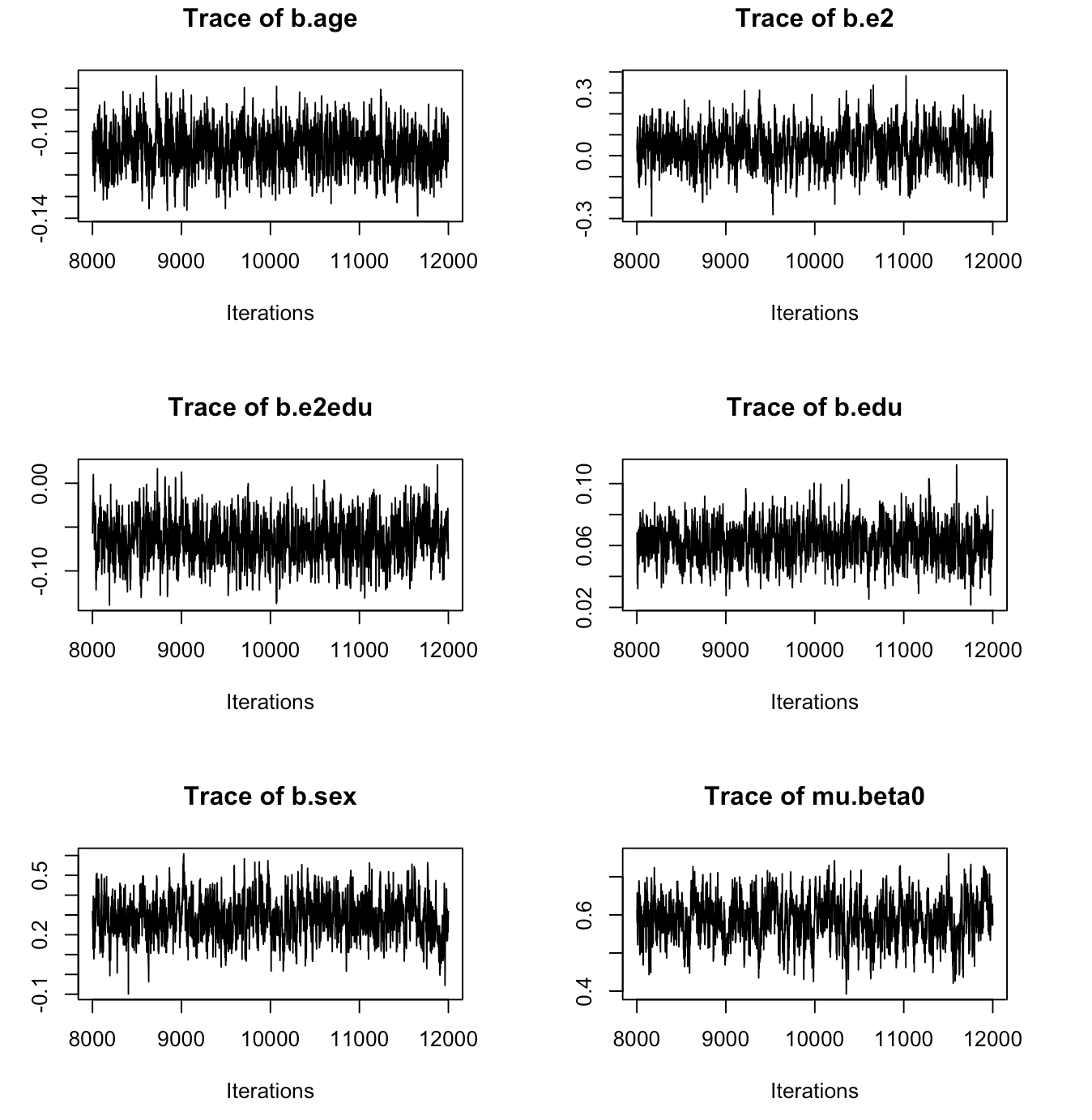


**Supplementary Figure 4**. Trace plot from the analysis of BIMC scores in carriers of *APOE* e4 and e3 alleles.


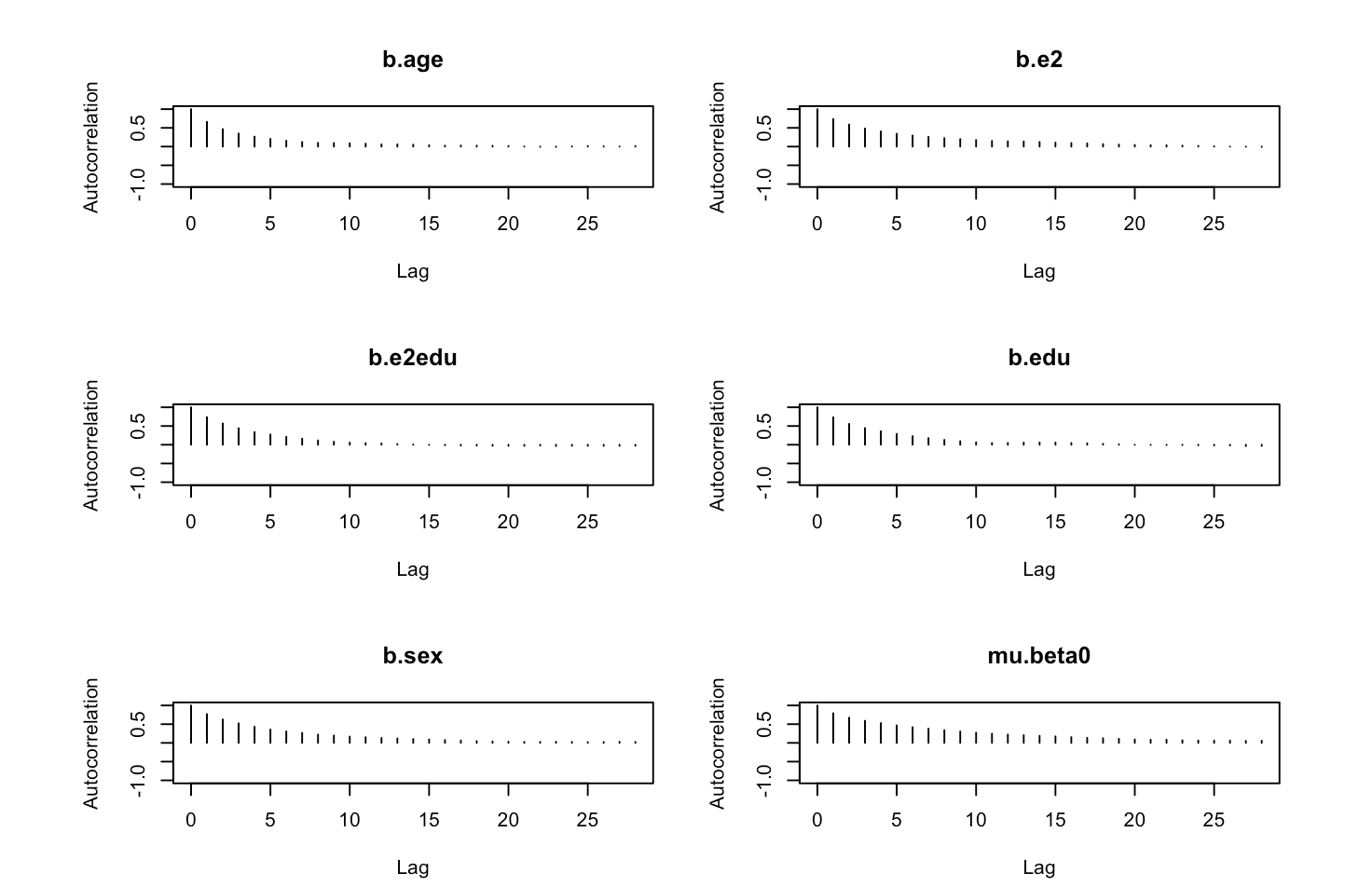


**Supplementary Figure 5**. Autocorrelation plot from the analysis of BIMC scores in carriers of *APOE* e4 and e3 alleles.


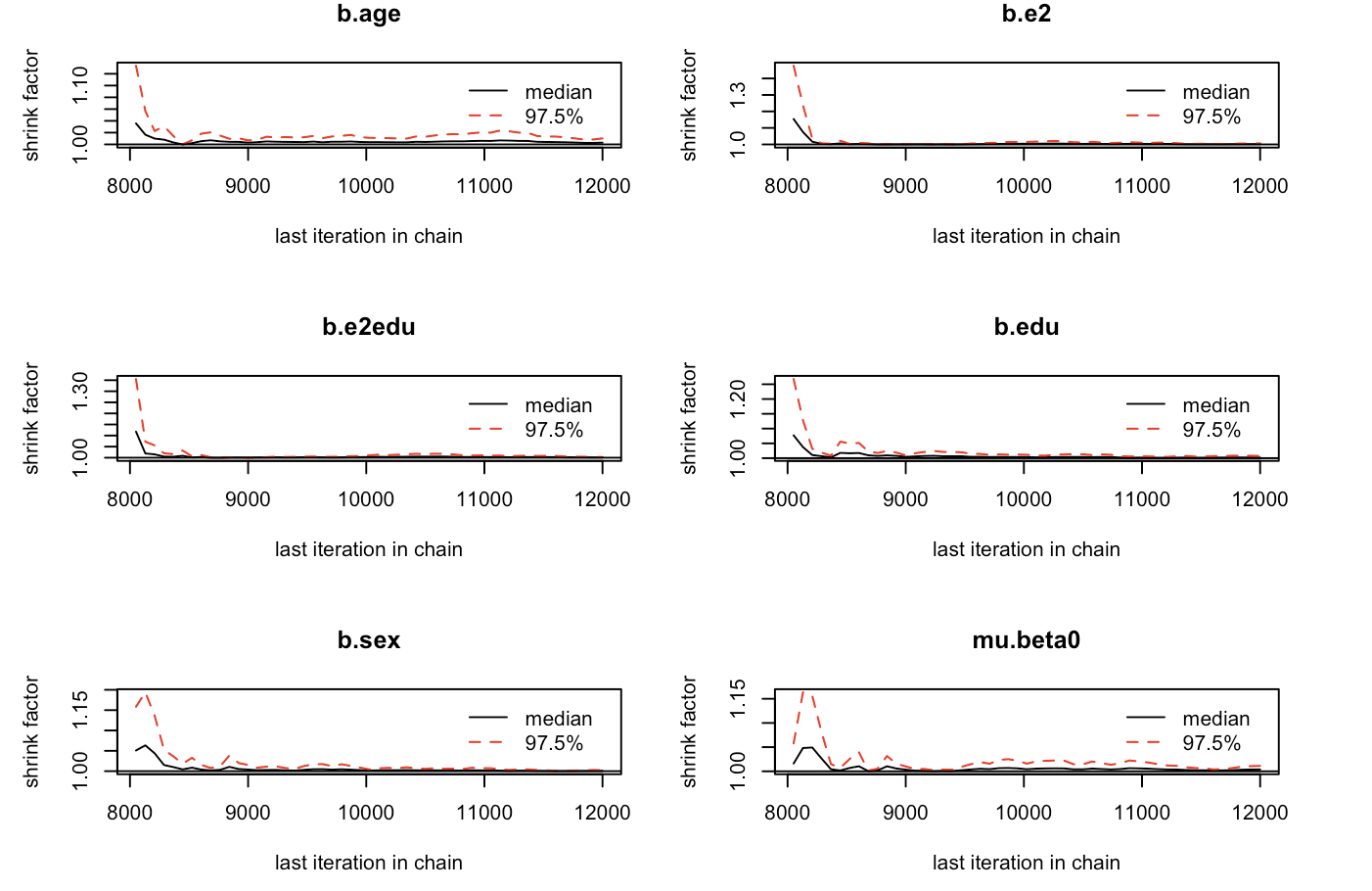


**Supplementary Figure 6.** Gelman plot from the analysis of BIMC scores in carriers of *APOE* e4 and e3 alleles.

## Supplementary Tables: sensitivity analysis

**Supplementary Table 2.** Parameter Estimates of the Sensitivity Analysis of BIMC Scores With Normal Prior Variance Changed to 100 in Carriers of *APOE* e2 and e3 Alleles in the New England Centenarian Study.

|  | **Estimates** | **2.5%CI** | **97.5%CI** | **SD** |
| --- | --- | --- | --- | --- |
| Intercept ($\mu_{b})$ | **0.587** | **0.489** | **0.695** | **0.053** |
| Age | **-0.108** | **-0.126** | **-0.089** | **0.009** |
| Sex, male | **0.288** | **0.107** | **0.485** | **0.098** |
| Education | **0.063** | **0.040** | **0.084** | **0.012** |
| *APOE*2 | 0.039 | -0.128 | 0.211 | 0.087 |
| *APOE*2*edu | **-0.064** | **-0.110** | **-0.018** | **0.024** |

**Supplementary Table 3.** Parameter Estimates of the Sensitivity Analysis of BIMC Scores With Normal Prior Variance Changed to 10 in Carriers of *APOE* e2 and e3 Alleles in the New England Centenarian Study.

|  | **Estimates** | **2.5%CI** | **97.5%CI** | **SD** |
| --- | --- | --- | --- | --- |
| Intercept ($\mu_{b})$ | **0.583** | **0.483** | **0.685** | **0.051** |
| Age | **-0.108** | **-0.125** | **-0.089** | **0.009** |
| Sex, male | **0.297** | **0.125** | **0.492** | **0.025** |
| Education | **0.062** | **0.040** | **0.085** | **0.011** |
| *APOE*2 | 0.041 | -0.127 | 0.208 | 0.085 |
| *APOE*2*edu | **-0.061** | **-0.109** | **-0.012** | **0.025** |

**Supplementary Table 4.** Parameter Estimates of the Sensitivity Analysis of BIMC Scores Where the Priors are the Gamma Distribution Changing the Mean to 1 and Variance to100 in Carriers of *APOE* e2 and e3 Alleles in the New England Centenarian Study.

|  | **Estimates** | **2.5%CI** | **97.5%CI** | **SD** |
| --- | --- | --- | --- | --- |
| Intercept ($\mu_{b})$ | **0.575** | **0.466** | **0.686** | **0.056** |
| Age | **-0.104** | **-0.123** | **-0.086** | **0.009** |
| Sex, male | **0.282** | **0.085** | **0.476** | **0.096** |
| Education | **0.061** | **0.036** | **0.084** | **0.011** |
| *APOE*2 | 0.045 | -0.133 | 0.086 | 0.089 |
| *APOE*2*edu | **-0.060** | **-0.110** | **-0.011** | **0.023** |

**Supplementary Table 5.** Parameter Estimates of the Sensitivity Analysis of BIMC Scores With Normal Prior Variance Changed to 10 in Carriers of *APOE* e4 and e3 Alleles in the New England Centenarian Study.

|  | **Estimates** | **2.5%CI** | **97.5%CI** | **SD** |
| --- | --- | --- | --- | --- |
| Intercept ($\mu_{b})$ | **0.648** | **0.540** | **0.755** | **0.056** |
| Age | **-0.117** | **-0.137** | **-0.097** | **0.010** |
| Sex, male | **0.219** | **0.017** | **0.422** | **0.105** |
| Education | **0.061** | **0.039** | **0.085** | **0.012** |
| *APOE*4 | -0.356 | -0.644 | -0.075 | 0.144 |
| *APOE*4*edu | **-0.079** | **-0.148** | **-0.011** | **0.034** |

**Supplementary Table 6.** Parameter Estimates of the Sensitivity Analysis of BIMC Scores With Normal Prior Variance Changed to 10 in Carriers of *APOE* e4 and e3 Alleles in the New England Centenarian Study.

|  | **Estimates** | **2.5%CI** | **97.5%CI** | **SD** |
| --- | --- | --- | --- | --- |
| Intercept ($\mu_{b})$ | **0.646** | **0.539** | **0.756** | **0.056** |
| Age | **-0.117** | **-0.139** | **-0.095** | **0.011** |
| Sex, male | **0.211** | **0.076** | **0.447** | **0.103** |
| Education | **0.061** | **0.038** | **0.085** | **0.012** |
| *APOE*4 | -0.346 | -0.628 | 0.065 | 0.144 |
| *APOE*4*edu | **-0.082** | **-0.152** | **-0.012** | **0.036** |

**Supplementary Table 7.** Parameter Estimates of the Sensitivity Analysis of BIMC Scores Where the Priors are the Gamma Distribution Changing the Mean to 1 and Variance to100 in Carriers of *APOE* e4 and e3 Alleles in the New England Centenarian Study.

|  | **Estimates** | **2.5%CI** | **97.5%CI** | **SD** |
| --- | --- | --- | --- | --- |
| Intercept ($\mu_{b})$ | **0.640** | **0.534** | **0.754** | **0.057** |
| Age | **-0.113** | **-0.134** | **-0.092** | **0.011** |
| Sex, male | **0.198** | **-0.019** | **0.414** | **0.108** |
| Education | **0.058** | **0.035** | **0.082** | **0.011** |
| *APOE*4 | **-0.334** | **-0.624** | **-0.051** | **0.147** |
| *APOE*4*edu | **-0.074** | **-0.147** | **-0.003** | **0.037** |

# Supplementary Codes

Below are the codes in R of the “rjags” setting of the Bayesian beta regression model:

"model{

for(i in 1:(Nset-1) ){

for(j in offset[i]:(offset[i+1]-1)){

Y[j] ~ dbeta(alpha[j], beta[j])

alpha[j] <- mu[j] * phi

beta[j] <- (1 - mu[j]) * phi

logit( mu[j] ) <- beta0[i]*indMea[i] + mu.beta0*(1-indMea[i]) + b.age*X.age[j] + b.sex*X.sex[j] + b.edu*X.edu[j] +

b.e2*( X.apoe[j,1]||X.apoe[j,2] ) + b.e2edu*(X.apoe[j,1]||X.apoe[j,2])*X.edu[j]

r[j] = Y[j] - mu[j]

}

beta0[i] ~ dnorm(mu.beta0, tau.beta0)

}

mu.beta0 ~ dnorm(0, 0.01)

b.age ~ dnorm(0, 0.01)

b.sex ~ dnorm(0, 0.01)

b.edu ~ dnorm(0, 0.01)

b.e2 ~ dnorm(0, 0.01)

b.e2edu ~ dnorm(0, 0.01)

tau.beta0 ~ dgamma(1, 1)

phi ~ dgamma(1, 1)

}"
